# Microvascular resistance reserve in relation to total and vessel-specific atherosclerotic burden

**DOI:** 10.1093/ehjci/jeae293

**Published:** 2024-11-12

**Authors:** Masahiro Hoshino, Ruurt A Jukema, Roel Hoek, Jorge Dahdal, Pieter Raijmakers, Roel Driessen, Michiel J Bom, Pepijn van Diemen, Jos Twisk, Ibrahim Danad, Tsunekazu Kakuta, Juhani Knuuti, Paul Knaapen

**Affiliations:** Departments of Cardiology, Amsterdam UMC, Vrije Universiteit Amsterdam, De Boelelaan 1117, 1081 HV Amsterdam, The Netherlands; Departments of Cardiology, Amsterdam UMC, Vrije Universiteit Amsterdam, De Boelelaan 1117, 1081 HV Amsterdam, The Netherlands; Departments of Cardiology, Amsterdam UMC, Vrije Universiteit Amsterdam, De Boelelaan 1117, 1081 HV Amsterdam, The Netherlands; Departments of Cardiology, Amsterdam UMC, Vrije Universiteit Amsterdam, De Boelelaan 1117, 1081 HV Amsterdam, The Netherlands; Department of Cardiovascular Diseases, Clínica Alemana de Santiago, Faculty of Medicine, Clínica Alemana Universidad del Desarrollo, Santiago, Chile; Radiology, Nuclear Medicine & PET Research, Amsterdam UMC, Vrije Universiteit Amsterdam, Amsterdam, The Netherlands; Departments of Cardiology, Amsterdam UMC, Vrije Universiteit Amsterdam, De Boelelaan 1117, 1081 HV Amsterdam, The Netherlands; Departments of Cardiology, Amsterdam UMC, Vrije Universiteit Amsterdam, De Boelelaan 1117, 1081 HV Amsterdam, The Netherlands; Departments of Cardiology, Amsterdam UMC, Vrije Universiteit Amsterdam, De Boelelaan 1117, 1081 HV Amsterdam, The Netherlands; Epidemiology & Data Science, Amsterdam UMC, Vrije Universiteit Amsterdam, Amsterdam, The Netherlands; Departments of Cardiology, Amsterdam UMC, Vrije Universiteit Amsterdam, De Boelelaan 1117, 1081 HV Amsterdam, The Netherlands; Department of Cardiology, University Medical Center Utrecht, Heidelberglaan 100, Utrecht 3584 CX, The Netherlands; Department of Cardiology, Tsuchiura Kyodo General Hospital, Ibaraki, Japan; Turku PET Centre, Turku University Hospital and University of Turku, Turku 20520, Finland; Clinical Physiology, Nuclear Medicine and PET, Turku University Hospital and University of Turku, Turku 20520, Finland; Departments of Cardiology, Amsterdam UMC, Vrije Universiteit Amsterdam, De Boelelaan 1117, 1081 HV Amsterdam, The Netherlands

**Keywords:** microvascular resistance • coronary atherosclerosis, [^15^O]H_2_O PET, fractional flow reserve

## Abstract

**Aims:**

The relationship between coronary artery atherosclerosis and microvascular resistance remains unclear. This study aims to clarify the relationship between total atherosclerotic and vessel-specific atherosclerotic burden and microvascular resistance reserve (MRR).

**Methods and results:**

In this *post hoc* analysis of the PACIFIC 1 trial, symptomatic patients without prior coronary artery disease (CAD) underwent [^15^O]H_2_O positron emission tomography, coronary computed tomography angiography (CCTA), and invasive fractional flow reserve (FFR). MRR was assessed across all three coronary branches, utilizing PET-derived coronary flow reserve and invasive FFR measurements. CCTA was used to assess patient and vessel-specific plaque volumes. Percentage atheroma volume (PAV) was defined as total plaque volume divided by vessel volume. The study included 142 patients (55% male, 57.5 ± 8.6 years) with 426 vessels with a mean MRR of 3.77 ± 1.64. While a significantly higher PAV was observed in the left anterior descending artery territory, MRR was similar across the three coronary branches. Generalized estimating equations without correction for cardiovascular risk factors identified that patient-specific PAV tertiles but not vessel-specific PAV tertiles were related to vessel-specific MRR. After correction for cardiovascular risk factors, compared with the first tertile of patient-specific PAV, the second tertile showed a vessel-specific MRR decrease of *β* = −0.362, *P* = 0.018, and the third tertile showed a decrease of *β* = −0.347, *P* = 0.024.

**Conclusion:**

In patients without prior CAD, patient-specific plaque burden was negatively associated to vessel-specific MRR; however, vessel-specific plaque burden was not related to vessel-specific MRR. Our findings suggest that the relation between atherosclerotic burden and an impaired microcirculatory function is of systemic origin.

## Graphical Abstract

**Graphical Abstract:**
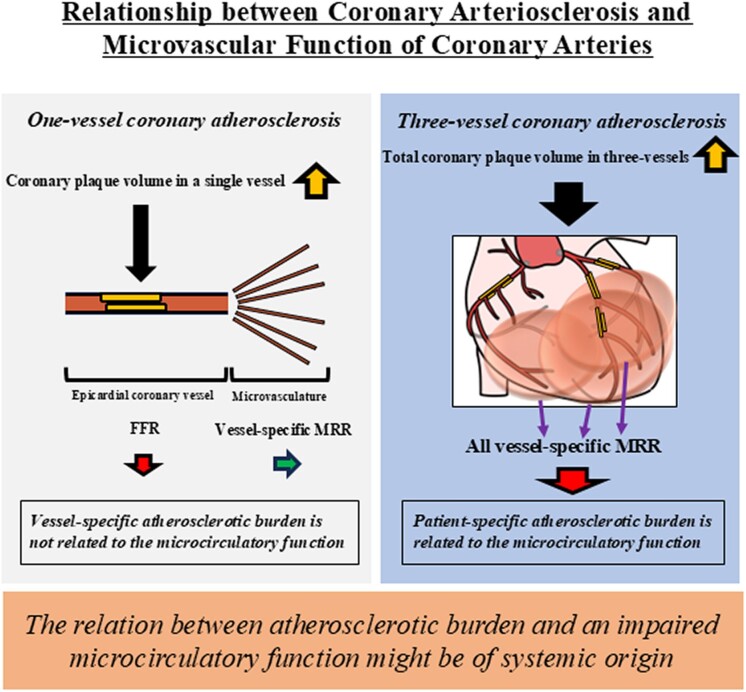

## Introduction

In recent years, there has been a significant focus on the relationship between coronary microcirculatory health and its clinical implications, particularly regarding symptoms and prognosis.^1,2^ The worsening of the microcirculatory system, influenced by risk factors associated with atherosclerosis, suggests a potential link with coronary atherosclerosis, yet this relationship remains a topic of debate.

The microcirculation system has an adaptive potential to maintain blood flow, even if epicardial artery stenoses are present.^3–5^ Additionally, the myocardium’s adaptive mechanisms, encompassing both local and systemic responses, underline the importance of how the epicardial and microcirculatory system relate.^6^ This highlights the necessity for a comprehensive approach in studying the relationship between microcirculation and coronary plaque, which takes into account not just individual vessels but the entire coronary system.

Furthermore, previous studies have primarily utilized coronary flow reserve (CFR) and the index of microcirculatory resistance (IMR) to explore the interaction between coronary plaque and microcirculation.^7–9^ However, these methodologies come with limitations. CFR is partially influenced by epicardial vessels, and IMR is subject to challenges such as myocardial mass^10^ and collateral circulation, as well inter-operator variability. To overcome these limitations, this study adopts the microvascular resistance reserve (MRR), a novel marker proposed by De Bruyne *et al*.^3^ for evaluating pure microvascular circulation without the need to account for myocardial mass or collateral circulation. By integrating invasive fractional flow reserve (FFR) and positron emission tomography (PET)-derived CFR measurements, our analysis aims to reveal the relationship between epicardial coronary atherosclerosis—marked by vessel-specific and patient-specific total coronary plaque burden—and MRR across the entire coronary system.

## Methods

### Patient selection

The study is a *post hoc* analysis of the Comparison of Coronary CT Angiography, SPECT, PET, and Hybrid Imaging for Diagnosis of Ischemic Heart Disease Determined by FFR (PACIFIC 1).^11^ The PACIFIC 1 trial was a prospective, single-centre, head-to-head comparative study from 2012 to 2017 at Amsterdam University Medical Center, location VU Medical Center in Amsterdam, the Netherlands (NCT01521468). All participants, with suspected obstructive coronary artery disease (CAD), completed a 2-week protocol including [^15^O]H_2_O PET and coronary computed tomography angiography (CCTA) prior to invasive coronary angiography (ICA) coupled with routine three-vessel invasive FFR examination. We focused on patients in whom MRR could be computed in all three coronary branches and in whom quantitative coronary plaque assessment on CCTA was available. The VUmc Medical Ethics Review Committee approved the study protocols and complied with the Declaration of Helsinki, with written informed consent obtained from all participants.

### PET procedure

PET scans were performed on a hybrid PET/CT equipment (Philips Gemini TF 64, Philips Healthcare, Best, the Netherlands). The scan procedure involved a dynamic 6 min scanning protocol that initiated simultaneously with an injection of 370 MBq [^15^O]H_2_O during resting and adenosine-induced hyperaemic conditions (140 µg/kg/min). Low-dose CT scans allowed for attenuation correction. Parametric images of quantitative hyperaemic MBF were created for each of the 17 segments of the left ventricle as per the American Heart Association model with standardized allocation of segments to the three vascular territories.^12^ Images were analysed using Carimas software (Turku PET Centre, University of Turku and Turku University Hospital, Turku, Finland). The participants were asked to abstain from caffeine or xanthine intake 24 h prior to the PET scan. Parametric MBF images were analysed. Regional hyperaemic myocardial blood flow (hMBF) was defined as mean hMBF of the entire vascular territory in the absence of a perfusion defect or as the mean hMBF of the perfusion defect (≥2 adjacent segments with a hMBF ≤ 2.3 mL/min/g) when present.^13^ CFR was defined as the ratio of hMBF to resting MBF (rMBF).

### ICA and physiological assessments

ICA was performed according to standard clinical protocols.^11^ Patients were instructed to refrain from the intake of xanthine or caffeine 24 h prior to the coronary angiography. All major coronary arteries (>2 mm) were routinely interrogated by FFR, irrespective of stenosis severity and imaging results. To induce maximal coronary hyperaemia, adenosine was administered intracoronary as a 150 μg bolus or intravenously (140 μg/kg/min). FFR was calculated as the ratio of mean distal intracoronary to aortic guiding pressure during hyperaemia. MRR was derived based on the framework by De Bruyne *et al*.^3^ by combining invasive FFR measurements and non-invasive PET flow measurements. The formula used in the current analysis is a quotient of CFR and FFR with the correction for the impact of haemodynamics, as follows:


$$\mathrm{MRR}=(\mathrm{CFR}/\mathrm{FFR})\;\times \;(P_{a,\mathrm{rest}}/P_{a,\mathrm{hyper}})$$

CFR indicates PET-derived CFR, FFR indicates pressure-wire derived FFR, and *P*_a,rest_ and *P*_a,hyper_ indicate mean aortic pressure during non-hyperaemic and maximal hyperaemic PET, respectively.

### CCTA

CT scans were performed using a 256-slice CT scanner (Philips Brilliance iCT, Philips Healthcare, Best, the Netherlands) with a tube current between 200 and 360 mAs at 120 kV. Respective CT parameters for the 256-slice entailed a section collimation of 128 × 0.625 mm and a gantry rotation time of 270 ms. Each patient received 800 mcg of sublingual nitroglycerine immediately prior to CCTA. If necessary, metoprolol was given prior to CCTA by oral or intravenous administration. For visualization of the coronary artery lumen, a bolus of iobitridol (Xenetix 350) was injected intravenously followed immediately by a saline chaser. Prospective electrocardiogram gating between 72 and 78% of the R-R interval was performed to reduce radiation dose.

### AI-QCT

A US Food and Drug Administration–approved artificial intelligence (AI)–based software approach (Cleerly Inc.) was used to analyse the CCTA.^14^ The AI-QCT software uses validated convolutional neural networks for image quality assessment, coronary segmentation, vessel contour determination, lumen wall evaluation, and plaque characterization and quantification. First, the algorithm produces a centreline, lumen, and outer vessel wall contours for every phase available. Subsequently, the algorithm selects the two optimal series for analysis for each coronary artery. Following automated segmentation and labelling of all coronary arteries, plaques are characterized and quantified based on Hounsfield unit attenuation. Finally, the AI analysis was supervised by a radiologic technologist for quality assurance review. Coronary segments with a diameter ≥ 1.5 mm were included for analysis. AI-QCT analyses were performed on a per segment basis using the modified 18-segment Society of Cardiovascular Computed Tomography model.^15^ Segments were evaluated for the presence of coronary atherosclerosis, defined as any tissue structure > 1 mm^2^ within the coronary artery wall that was differentiated from the surrounding epicardial tissue, epicardial fat, or the vessel lumen itself. Plaque volumes (mm^3^) were calculated for each coronary lesion and then summated to compute the total plaque volume for the entire segment. Vessel-specific plaque volumes were normalized to the vessel volume to account for variation in coronary artery volume, calculated as vessel-specific plaque volume/vessel-specific volume × 100%. These normalized volumes were reported as percentage atheroma volume (PAV). Calcified plaque volume (CPV)/vessel-specific volume and non-CPV (NCPV)/vessel-specific volume were also reported as PCPV (percentage CPV) and PNCPV (percentage NCPV). To match plaque findings with invasive FFR measurements, plaque and vessel volumes were summoned from the position of the aortic pressure sensor until the distal pressure sensor. If image quality was insufficient or artefacts hindered analysis, only the slices with insufficient quality were excluded from the analysis. The AI-QCT algorithm has been validated against expert CT readers, quantitative coronary angiography, and intravascular ultrasound.^14,16–18^ Furthermore, AI-QCT-derived plaque staging showed important prognostic value in addition to clinical risk factors, coronary calcium score, diameter stenosis, and CAD-RADS.^19^ The radiologic technologists who performed quality assurance review were blinded to the results of the invasive coronary pressure measurements.

### Statistical analysis

Continuous variables were presented as mean ± standard deviation or median with interquartile range, based on their distribution. Categorical variables were expressed as frequencies and percentages. The Kruskal–Wallis test was used to compare FFR, PAV, PCPV, and PNCPV among the three coronary branches, whereas the analysis of variance was used to compare vessel-specific MRR and CFR. Given that plaque volumes were non-normally distributed, Spearman’s correlation was used to assess the correlations between vessel-specific MRR and PAV, PCPV, and PNCPV for each of the three coronary branches. The correlation between mean MRR across the three coronary branches and total plaque volume was also analysed using Spearman’s correlation coefficient. To correct for cardiovascular risk factors and for multiple vessels within a patient, generalized estimating equations (GEEs) were used to assess the relation between plaque volumes and vessel-specific MRR. Plaque burdens were introduced to the GEE using tertiles. Including plaque burden as a continuous variable in the GEE models was unsuitable because the relationship between plaque burden and MRR is non-linear. Logarithmic transformation was also unsuitable because multiple vessels had plaque burden values of zero. Therefore, the GEE models were constructed based on the tertiles of PAV, PCPV, and PNCPV. Significant predictors of vessel-specific MRR in the univariable analysis (*P* < 0.05) were included into the multivariable model.

A two-sided *P* < 0.05 was considered statistically significant. Statistical analyses were performed using R version 4.3.1 (R Foundation for Statistical Computing, Vienna, Austria).

## Results

Among the 208 patients enrolled in the PACIFIC 1 study, 179 (86%) had three-vessel FFR, and 164 (79%) had three-vessel CFR available. As such, 144 patients had three-vessel MRR, representing 69% of the total cohort. Another two patients were excluded due to uninterpretable CCTA. The final analysis included 142 patients (see Supplementary data online, *Figure S1*).

**Figure 1:**
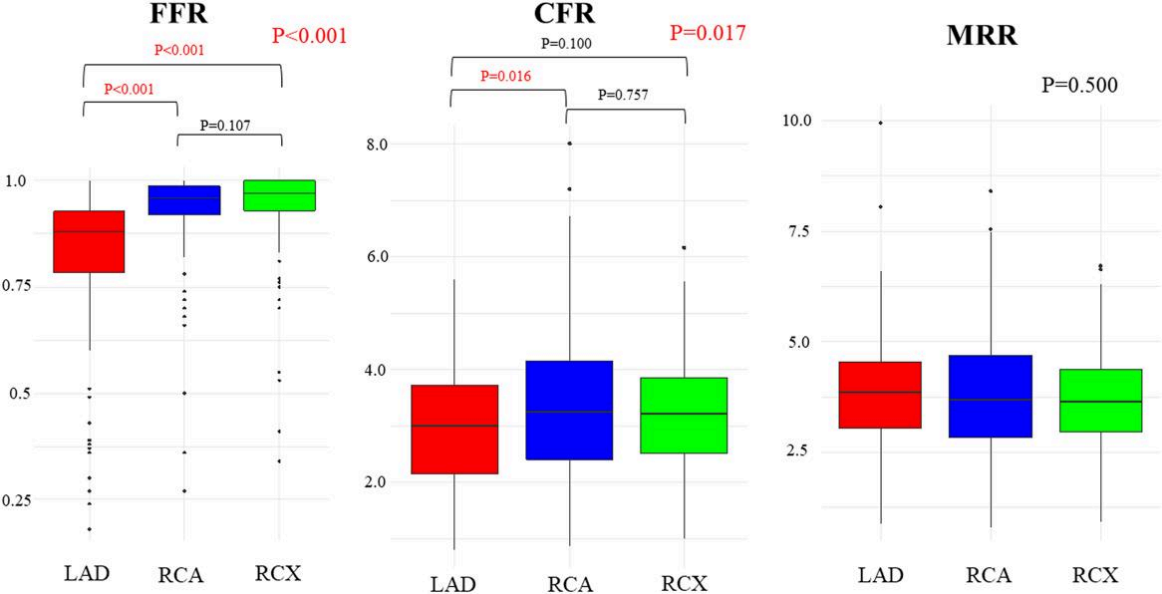
Comparative analysis of FFR, CFR, and MRR across coronary branches. The comparison of FFR, CFR, and MRR across LAD, RCA, and RCX arteries. CFR, coronary flow reserve; FFR, fractional flow reserve; MRR, microvascular resistance reserve; LAD, left anterior descending artery; RCA, right coronary artery; RCX, right circumflex artery.

The patients’ mean age was 57.5 ± 8.6 years, and 78 (54.9%) were male. Further patient characteristics are described in *Table 1*. Vessel-specific characteristics are displayed in *Table 2*. Overall, FFR and CFR were significantly lower in left anterior descending artery (LAD) region compared with the other areas. However, MRR was similar among the three coronary branches (*Figure 1*).

**Table 1:** Baseline characteristics

	All patients *n* = 142
Characteristics	
Male	78 (54.9%)
Age, years	57.5 ± 8.6
BMI, kg/m^2^	26.7 ± 3.7
Cardiovascular risk factors	
Diabetes mellitus	20 (14.1%)
Hypertension	67 (47.2%)
Hypercholesterolaemia	51 (35.9%)
History of smoking	27 (19.0%)
Family history of CAD	77 (54.2%)
Medication	
Antiplatelet therapy	123 (86.6%)
β-Blocker	89 (62.7%)
Calcium channel blocker	39 (27.5%)
ACE-inhibitor	28 (19.7%)
ARB	25 (17.6%)
Statin	107 (75.4%)
Long-acting nitrate	16 (11.3%)

BMI, body mass index; CAD, coronary artery disease; ACE, angiotensin-converting enzyme; ARB, angiotensin II receptor blockers.

**Table 2:** Vessel characteristics

	All vessels *n* = 142 (426 vessels)				
	Overall	LAD	RCA	RCX	*P* value
FFR	0.94 (0.88–0.98)	0.88 (0.78–0.93)	0.96 (0.92–0.99)	0.97 (0.93–1.00)	<0.001
CFR	3.20 ± 1.54	2.97 ± 1.58	3.36 ± 1.75	3.26 ± 1.36	0.017
MRR	3.77 ± 1.64	3.85 ± 1.50	3.79 ± 1.88	3.67 ± 1.41	0.500
PAV (%)	8.0 (2.3–15.5)	11.5 (2.6–24.9)	2.8 (0.9–12.3)	4.6 (1.6–13.6)	<0.001
PCPV (%)	2.3 (0.1–6.3)	2.9 (0–9.5)	0.1 (0–4.0)	0.6 (0–4.8)	<0.001
PNCPV (%)	4.3 (1.7–9.3)	6.2 (1.9–12.9)	2.1 (0.9–7.2)	3.6 (1.4–9.0)	<0.001

FFR, fractional flow reserve; CFR, coronary flow reserve; MRR, microvascular resistance reserve; PAV, percentage atheroma volume; PCPV, percentage calcified plaque volume; PNCPV, percentage non-calcified plaque volume.

**Figure 2:**
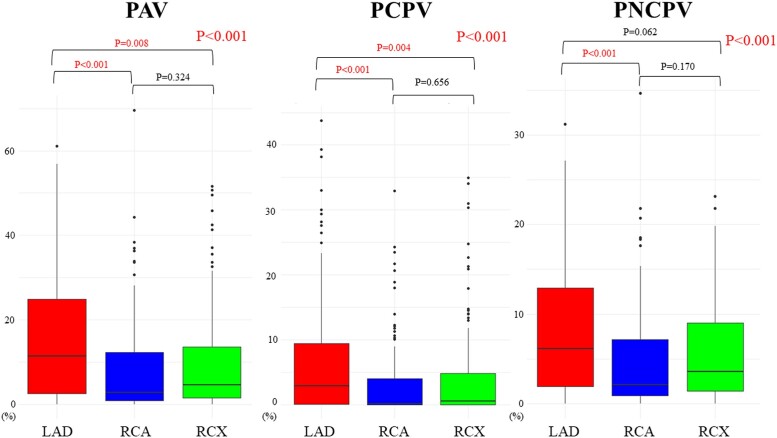
Comparative analysis of vessel-specific plaque volumes/vessel volumes across coronary branches. The comparison of vessel-specific PAV, PCPV, and PNCPV across LAD, RCA, and RCX arteries. PAV, percentage atheroma volume; PCPV, percentage calcified plaque volume; PNCPV, percentage non-calcified plaque volume. Other abbreviations are in *Figure 1*.

In patients with single-vessel obstructive CAD, obstructive CAD (FFR ≤ 0.80) was most frequently observed in the LAD (27 patients, 19.0%), followed by right coronary artery (RCA) (2 patients, 1.4%) and right circumflex artery (RCX) (3 patients, 2.1%). In cases of two-vessel disease, the LAD/RCA combination was found in five patients (3.5%), LAD/RCX in five patients (3.5%), and RCA/RCX in one patient (0.7%). Three-vessel disease was observed in two patients (1.4%). PAV and PCPV were particularly higher in the LAD territory in comparison with other target vessel areas. On the other hand, PAV, PCPV, and PNCPV values were similar between RCA and RCX (*Figure 2*).

### Correlation analysis: FFR, MRR, and coronary plaque volumes

**Figure 3:**
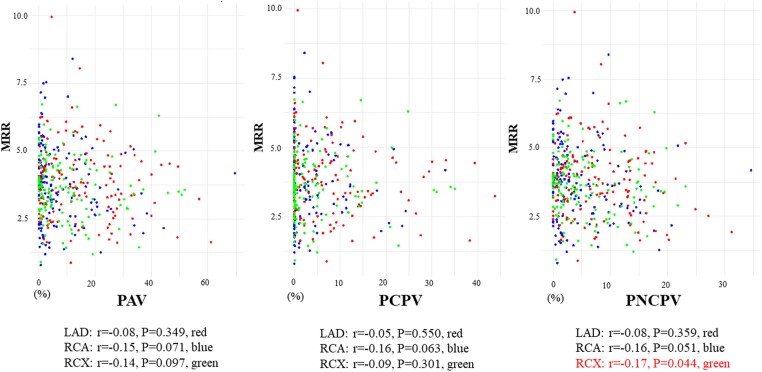
Correlation of vessel-specific plaque volumes to vessel-specific MRR. The correlations between vessel-specific PAV, PCPV, and PNCPV to vessel-specific MRR. Each subplot represents a different plaque type, with scatter plots illustrating the relationship between plaque volume and MRR within individual coronary arteries. Abbreviations are in *Figures 1* and *2*.

**Figure 4:**
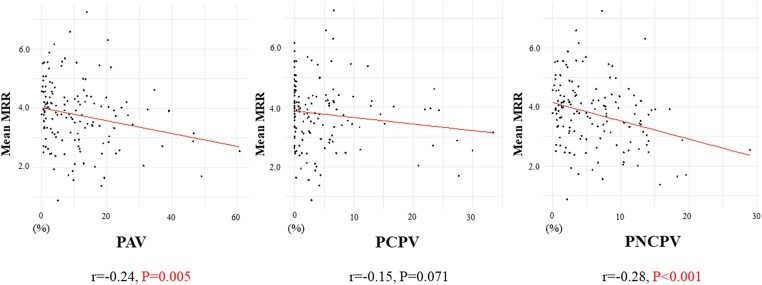
Correlation between mean MRR and patient-specific plaque volumes. The correlations between patient-specific PAV, PCPV, and PNCPV to mean MRR. Each subplot represents a different plaque type, with scatter plots illustrating the relationship between summed plaque volume and mean MRR within three coronary arteries. Abbreviations are in *Figures 1* and *2*.

FFR and MRR were not correlated in a vessel-specific analysis (see Supplementary data online, *Figure S2*). PAV, PCPV, and PNCPV were not correlated to vessel-specific MRR for the LAD and RCA territories; however, a weak correlation between vessel-specific MRR and PNCPV was observed in the RCX (*Figure 3*). On a per-patient basis using mean MRR of the three coronary territories, a negative correlation was observed with PAV (*r* = −0.24) and PNCPV (*r* = −0.28), whereas the correlation with PCPV (*r* = −0.15) was not statistically significant (*Figure 4*).

### Impact of coronary plaque volume on MRR

Linear regression analysis using GEE indicated that in a univariable analysis, sex, smoking status, and the patient-specific total plaque (PAV, PCPV, and PNCPV) were associated with vessel-specific MRR.

In contrast, vessel-specific plaque volumes were not significantly related with vessel-specific MRR (*Table 3*). In the multivariable analysis, male sex (*β* = −0.379, *P* = 0.004), smoking (*β* = −0.529, *P* < 0.001), and patient-specific PAV tertiles independently predicted vessel-specific MRR (*Table 3*). Compared with the first tertile of patient-specific PAV, the second tertile showed a vessel-specific MRR decrease (*β* = −0.362, *P* = 0.018), and the third tertile showed a decrease (*β* = −0.347, *P* = 0.024).

**Table 3:** GEEs for vessel-specific MRR

	Univariable analysis		Multivariable analysis	
	*β*	*P* value	*β*	*P* value
Age	−0.009	0.250		
Male	−0.498	<0.001	−0.379	0.004
BMI	−0.002	0.910		
Diabetes mellitus	−0.231	0.200		
Hypertension	−0.236	0.063		
Current smoker	−0.585	<0.001	−0.529	<0.001
Vessel-specific plaque volume/vessel volume				
PAV				
Quartile 1	Reference			
Quartile 2 vs. 1	−0.078	0.621		
Quartile 3 vs. 1	−0.249	0.093		
PCPV				
Quartile 1	Reference			
Quartile 2 vs. 1	−0.308	0.052		
Quartile 3 vs. 1	−0.263	0.080		
PNCPV				
Quartile 1	Reference			
Quartile 2 vs. 1	0.092	0.544		
Quartile 3 vs. 1	−0.278	0.076		
Patient-specific plaque volume/vessel volume				
PAV				
Quartile 1	Reference		Reference	
Quartile 2 vs. 1	−0.486	0.001	−0.362	0.018
Quartile 3 vs. 1	−0.465	0.002	−0.347	0.024
PCPV				
Quartile 1	Reference			
Quartile 2 vs. 1	−0.632	<0.001		
Quartile 3 vs. 1	−0.240	0.120		
PNCPV				
Quartile 1	Reference			
Quartile 2 vs. 1	−0.240	0.122		
Quartile 3 vs. 1	−0.560	<0.001		

BMI, body mass index; MRR, microvascular resistance reserve; PAV, percentage atheroma volume; PCPV, percentage calcified plaque volume; PNCPV, percentage non-calcified plaque volume.

### Relationship between risk factor management and MRR

Even when LDL cholesterol was well controlled (<70 mg/dL) or BMI was ≤30 kg/m², there was no significant difference in mean MRR values (LDL: *P* = 0.547; BMI: *P* = 0.451). MRR was reduced in current smokers, with mean MRR values of 3.29 ± 1.00 for current smokers, 3.94 ± 1.12 for former smokers, and 3.88 ± 1.11 for non-smokers (*P* = 0.040) (see Supplementary data online, *Figure S3*).

## Discussion

Our study provides a unique analysis of the complex interaction between coronary artery atherosclerosis and the myocardial microcirculation in patients without prior myocardial infarction by examining the relationship between coronary plaque burden and MRR. This study found that vessel-specific plaque burden was not related to MRR, whereas patient-specific PAV was negatively correlated to MRR. Moreover, although there is no direct correlation between the microcirculation function and coronary plaque in individual coronary arteries, an increase in cumulative plaque burden, particularly non-calcified plaque burden, across all three coronary arteries was significantly associated with an impaired microcirculatory function.

The formation of coronary plaque is a lipid-driven inflammatory disease within the arterial wall. Moreover, the accumulation of coronary plaque might also have focal aspects, potentially because of focal factors like endothelial shear stress.^20,21^ These aspects opened the debate whether the microcirculatory function predominantly is a vessel-specific feature or a patient-specific feature and how these aspects of epicardial atherosclerosis relate to the microcirculatory function. On the other hand, coronary risk factors such as age, hypertension, diabetes, and smoking impact both microvascular dysfunction and the formation and progression of coronary plaques, suggesting a potential correlation between worsening coronary plaques and the progression of microcirculatory dysfunction.^5^ In this study, current smokers exhibited a lower MRR compared with former smokers and non-smokers, suggesting that smoking contributes to microvascular dysfunction independently of the influence of coronary plaque burden. Our findings imply that smoking cessation may improve microvascular function. These findings highlight the importance of addressing smoking, a modifiable risk factor, in the management of patients with CAD.

If a coronary stenosis leads to a decrease in FFR, microcirculation undergoes compensatory dilation to supplement the reduced blood flow.^4,22^ Therefore, the relationship between the deterioration of microcirculation and coronary plaque is not necessarily directly correlated. Moreover, the compensatory mechanisms of the microcirculation and collateral pathways between coronary branches further complicate this relationship.^5,23^ The absence of a direct relationship between vessel-specific plaque burden and MRR in its corresponding territory may be influenced by compensatory blood flow processes from the other coronary arteries. Indeed, the cumulative impact of summed coronary plaque from the three coronary arteries was significantly associated to a decreased microcirculatory function. This underscores the complexity of the assessment of the relationship between coronary plaque and microcirculatory function using a single coronary artery branch, highlighting the need for a more comprehensive approach that considers the interplay between all branches in understanding the effects of atherosclerosis on microcirculation. The interplay between total epicardial plaque burden and MRR is independent of cardiovascular risk factors and is essential for understanding the pathophysiology of ischaemic heart disease and development of treatment strategies.

A number of studies suggested a relation between microvascular dysfunction and vessel-specific coronary plaque formation using CFR and IMR.^7,8,24^ Compared with those studies, the population in this study with a history of no heart disease indicates a relatively low level of atherosclerosis. Depending on the degree of atherosclerosis, it is possible to experience effective compensatory coronary blood flow through microcirculatory adaption. In chronic ischaemic conditions, the initial compensatory response of the microcirculation undergoes functional and structural changes over time. These physiological changes further complicate the comprehensive analysis of the microcirculatory function and include altered myogenic responses, microvascular vasoconstriction, and a potential reduction in vasodilatory capacity.^25^ Such alterations can ultimately promote a decrease in myocardial perfusion and the loss of myocytes, suggesting that chronic atherosclerosis is a significant factor in causing microcirculatory dysfunction.^5,25^ The microcirculation may be affected differently by the stage of atherosclerosis in relation to coronary plaque involvement. During the early stages of atherosclerosis, we found that the overall atherosclerotic burden negatively impacted MRR independent of traditional risk factors, suggesting a systemic rather than localized effect on microcirculatory function.

## Limitation

Some limitations should be acknowledged. Firstly, this was a cross-sectional study with a limited sample size making it difficult to establish causality. Secondly, while prior studies suggested that FFR and MRR might be independent of vessel-specific atherosclerotic burden,^3^ MRR might not be entirely independent of the status of epicardial disease. Thirdly, this study did not entail endothelial function tests for assessing arteriolar dysregulation in the coronary microcirculation. Fourth, segments evaluated by PET for CFR and by invasive wire for FFR may not perfectly align, in contrast to coronary flow assessment using a wire. Fifthly, while we did not obtain invasive flow and resistance values typically acquired through invasive measurements alone, the combination of FFR and PET allowed for a precise evaluation of microvascular function. This approach yields valuable insights into the pathophysiological mechanisms of ischaemic heart disease, but it may not be feasible for widespread clinical use. Lastly, although we attempted to examine the relationship between MRR and clinical outcomes, only four patients (3%) experienced major adverse events such as death (*n* = 1) or myocardial infarction (*n* = 3) during long-term follow-up (1680 ± 331 days), which was insufficient for prognostic analysis. Further studies are needed to investigate how MRR and coronary plaque burden relate to long-term prognosis.

## Conclusion

Our study demonstrated an independent relation between total atherosclerotic plaque volume and an impaired microcirculatory function. The relation between atherosclerotic burden and impaired microcirculatory function appeared stronger on a per-patient level than for vessel-specific parameters. Our findings indicate that the relation between atherosclerotic burden and an impaired microcirculatory function might be of systemic origin.

## Supplementary data

Supplementary data are available at *European Heart Journal - Cardiovascular Imaging* online.

## Supplementary Material

jeae293_Supplementary_Data

## Data Availability

Raw data are available upon reasonable request to the corresponding author.

## References

[1] Schroder J, Michelsen MM, Mygind ND, Suhrs HE, Bove KB, Bechsgaard DF et al Coronary flow velocity reserve predicts adverse prognosis in women with angina and no obstructive coronary artery disease: results from the iPOWER study. Eur Heart J 2021;42:228–39. 33477168 10.1093/eurheartj/ehaa944

[2] Boerhout CKM, Lee JM, de Waard GA, Mejia-Renteria H, Lee SH, Jung JH et al Microvascular resistance reserve: diagnostic and prognostic performance in the ILIAS registry. Eur Heart J 2023;44:2862–9. 37350567 10.1093/eurheartj/ehad378 PMC10406337

[3] De Bruyne B, Pijls NHJ, Gallinoro E, Candreva A, Fournier S, Keulards DCJ et al Microvascular resistance reserve for assessment of coronary microvascular function: JACC technology corner. J Am Coll Cardiol 2021;78:1541–9. 34620412 10.1016/j.jacc.2021.08.017

[4] Duncker DJ, Koller A, Merkus D, Canty JM Jr Regulation of coronary blood flow in health and ischemic heart disease. Prog Cardiovasc Dis 2015;57:409–22. 25475073 10.1016/j.pcad.2014.12.002 PMC5856234

[5] Merkus D, Muller-Delp J, Heaps CL. Coronary microvascular adaptations distal to epicardial artery stenosis. Am J Physiol Heart Circ Physiol 2021;320:H2351–70. 33961506 10.1152/ajpheart.00992.2020 PMC8289363

[6] Seiler C, Stoller M, Pitt B, Meier P. The human coronary collateral circulation: development and clinical importance. Eur Heart J 2013;34:2674–82. 23739241 10.1093/eurheartj/eht195

[7] Hoshino M, Yang S, Sugiyama T, Zhang J, Kanaji Y, Hamaya R et al Characteristic findings of microvascular dysfunction on coronary computed tomography angiography in patients with intermediate coronary stenosis. Eur Radiol 2021;31:9198–210. 34009414 10.1007/s00330-021-07909-7

[8] Usui E, Yonetsu T, Kanaji Y, Hoshino M, Yamaguchi M, Hada M et al Optical coherence tomography-defined plaque vulnerability in relation to functional stenosis severity and microvascular dysfunction. JACC Cardiovasc Interv 2018;11:2058–68. 30336810 10.1016/j.jcin.2018.07.012

[9] Hoshino M, Usui E, Sugiyama T, Kanaji Y, Yonetsu T, Kakuta T. Prevalence of OCT-defined high-risk plaque in relation to physiological characteristics by fractional flow reserve and coronary flow reserve. Rev Esp Cardiol (Engl Ed) 2020;73:331–2. 31672561 10.1016/j.rec.2019.09.008

[10] Echavarria-Pinto M, van de Hoef TP, Nijjer S, Gonzalo N, Nombela-Franco L, Ibanez B et al Influence of the amount of myocardium subtended to a coronary stenosis on the index of microcirculatory resistance. Implications for the invasive assessment of microcirculatory function in ischaemic heart disease. EuroIntervention 2017;13:944–52. 28485281 10.4244/EIJ-D-16-00525

[11] Danad I, Raijmakers PG, Driessen RS, Leipsic J, Raju R, Naoum C et al Comparison of coronary CT angiography, SPECT, PET, and hybrid imaging for diagnosis of ischemic heart disease determined by fractional flow reserve. JAMA Cardiol 2017;2:1100–7. 28813561 10.1001/jamacardio.2017.2471 PMC5710451

[12] Cerqueira MD, Weissman NJ, Dilsizian V, Jacobs AK, Kaul S, Laskey WK et al Standardized myocardial segmentation and nomenclature for tomographic imaging of the heart. A statement for healthcare professionals from the Cardiac Imaging Committee of the Council on Clinical Cardiology of the American Heart Association. Int J Cardiovasc Imaging 2002;18:539–42. 12135124

[13] Danad I, Uusitalo V, Kero T, Saraste A, Raijmakers PG, Lammertsma AA et al Quantitative assessment of myocardial perfusion in the detection of significant coronary artery disease: cutoff values and diagnostic accuracy of quantitative [(15)O]H2O PET imaging. J Am Coll Cardiol 2014;64:1464–75. 25277618 10.1016/j.jacc.2014.05.069

[14] Griffin WF, Choi AD, Riess JS, Marques H, Chang HJ, Choi JH et al AI evaluation of stenosis on coronary CT angiography, comparison with quantitative coronary angiography and fractional flow reserve: a CREDENCE trial substudy. JACC Cardiovasc Imaging 2023;16:193–205. 35183478 10.1016/j.jcmg.2021.10.020

[15] Leipsic J, Abbara S, Achenbach S, Cury R, Earls JP, Mancini GJ et al SCCT guidelines for the interpretation and reporting of coronary CT angiography: a report of the Society of Cardiovascular Computed Tomography Guidelines Committee. J Cardiovasc Comput Tomogr 2014;8:342–58. 25301040 10.1016/j.jcct.2014.07.003

[16] Choi AD, Marques H, Kumar V, Griffin WF, Rahban H, Karlsberg RP et al CT evaluation by artificial intelligence for atherosclerosis, stenosis and vascular morphology (CLARIFY): a multi-center, international study. J Cardiovasc Comput Tomogr 2021;15:470–6. 34127407 10.1016/j.jcct.2021.05.004

[17] Omori H, Matsuo H, Fujimoto S, Sobue Y, Nozaki Y, Nakazawa G et al Determination of lipid-rich plaques by artificial intelligence-enabled quantitative computed tomography using near-infrared spectroscopy as reference. Atherosclerosis 2023;386:117363. 37944269 10.1016/j.atherosclerosis.2023.117363

[18] Jonas RA, Weerakoon S, Fisher R, Griffin WF, Kumar V, Rahban H et al Interobserver variability among expert readers quantifying plaque volume and plaque characteristics on coronary CT angiography: a CLARIFY trial sub-study. Clin Imaging 2022;91:19–25. 35986973 10.1016/j.clinimag.2022.08.005

[19] Nurmohamed NS, Bom MJ, Jukema RA, de Groot RJ, Driessen RS, van Diemen PA et al AI-guided quantitative plaque staging predicts long-term cardiovascular outcomes in patients at risk for atherosclerotic CVD. JACC Cardiovasc Imaging 2024;17:269–80. 37480907 10.1016/j.jcmg.2023.05.020

[20] Choi G, Lee JM, Kim HJ, Park JB, Sankaran S, Otake H et al Coronary artery axial plaque stress and its relationship with lesion geometry: application of computational fluid dynamics to coronary CT angiography. JACC Cardiovasc Imaging 2015;8:1156–66. 26363834 10.1016/j.jcmg.2015.04.024

[21] Yahagi K, Kolodgie FD, Otsuka F, Finn AV, Davis HR, Joner M et al Pathophysiology of native coronary, vein graft, and in-stent atherosclerosis. Nat Rev Cardiol 2016;13:79–98. 26503410 10.1038/nrcardio.2015.164

[22] Weil BR, Suzuki G, Canty JM Jr Transmural variation in microvascular remodeling following percutaneous revascularization of a chronic coronary stenosis in swine. Am J Physiol Heart Circ Physiol 2020;318:H696–705. 32056445 10.1152/ajpheart.00502.2019 PMC7099450

[23] Keulards DCJ, Karamasis GV, Alsanjari O, Demandt JPA, Van't Veer M, Zelis JM et al Recovery of absolute coronary blood flow and microvascular resistance after chronic total occlusion percutaneous coronary intervention: an exploratory study. J Am Heart Assoc 2020;9:e015669. 32316813 10.1161/JAHA.119.015669 PMC7428549

[24] Dhawan SS, Corban MT, Nanjundappa RA, Eshtehardi P, McDaniel MC, Kwarteng CA et al Coronary microvascular dysfunction is associated with higher frequency of thin-cap fibroatheroma. Atherosclerosis 2012;223:384–8. 22766333 10.1016/j.atherosclerosis.2012.05.034

[25] Sorop O, Merkus D, de Beer VJ, Houweling B, Pistea A, McFalls EO et al Functional and structural adaptations of coronary microvessels distal to a chronic coronary artery stenosis. Circ Res. 2008;102:795–803. 18292598 10.1161/CIRCRESAHA.108.172528

