# Supplementary figures and images for: Microvascular resistance reserve in relation to total and vessel-specific atherosclerotic burden
Source: Eur Heart J Cardiovasc Imaging. 2024 Nov 12;26(3):481–8. doi: 10.1093/ehjci/jeae293 (PMC11879247; doi:10.1093/ehjci/jeae293)

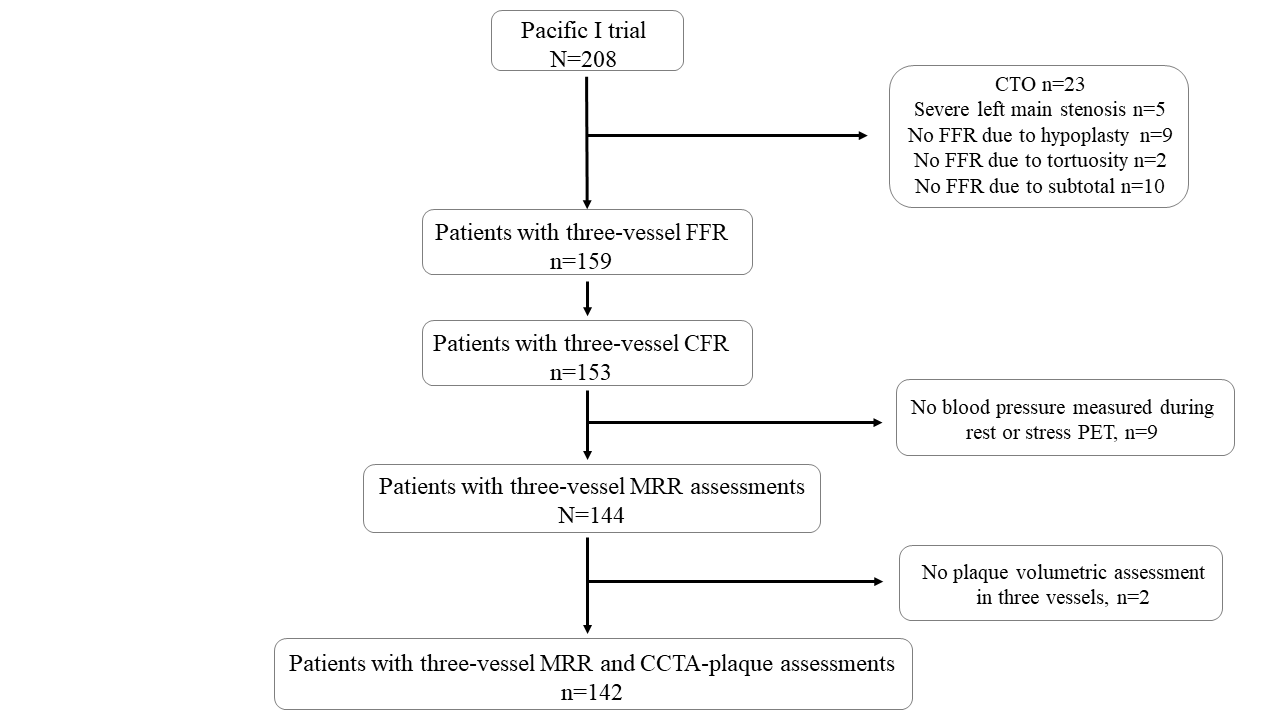

Supplement: jeae293_Supplementary_Data [file jeae293_supplementary_data.zip › Supplemental Figure 1_new.tif]

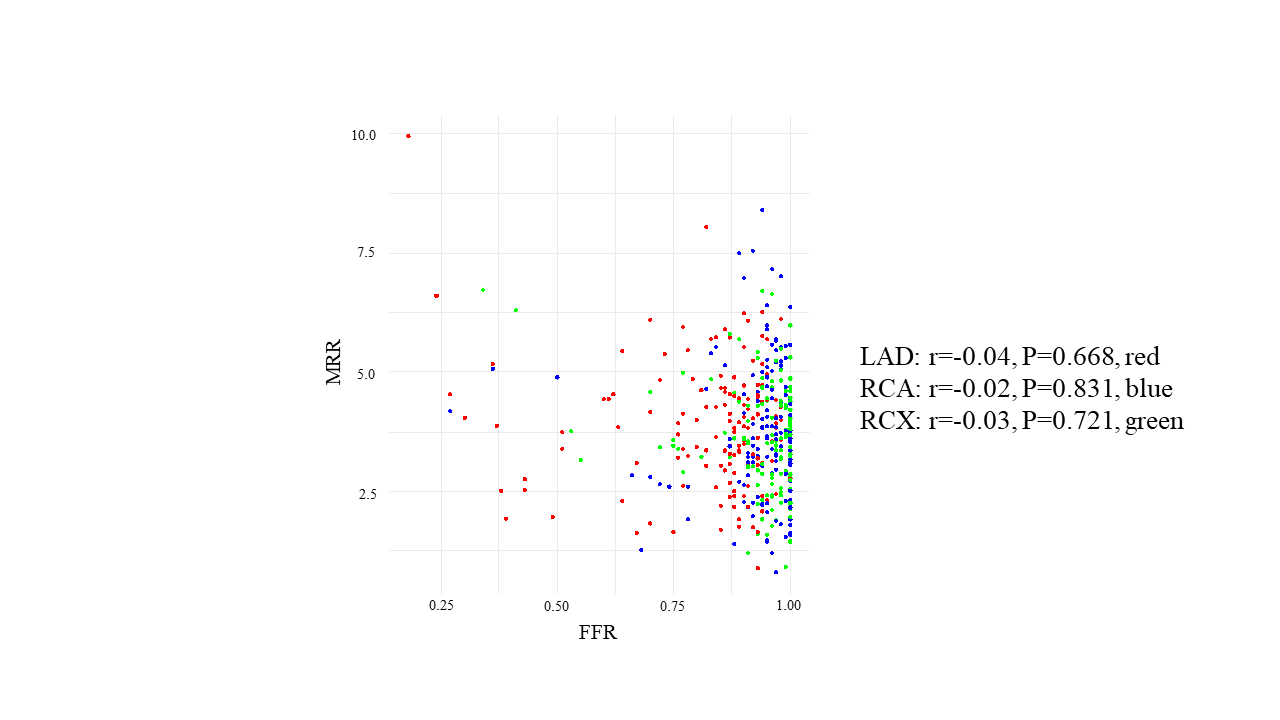

Supplement: jeae293_Supplementary_Data [file jeae293_supplementary_data.zip › Supplemental Figure 2_new.tif]

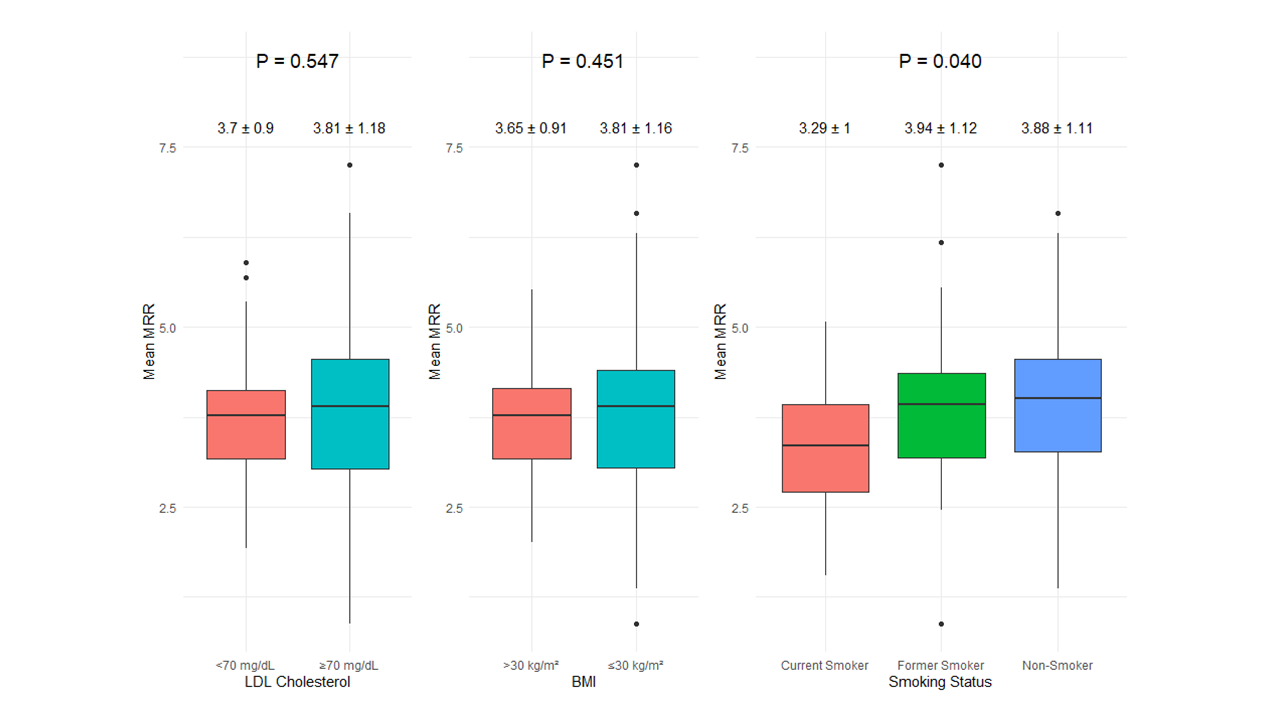

Supplement: jeae293_Supplementary_Data [file jeae293_supplementary_data.zip › Supplemental Figure 3.tif]
